# ZIF-8 as a promising drug delivery system for benznidazole: development, characterization, in vitro dialysis release and cytotoxicity

**DOI:** 10.1038/s41598-020-73848-w

**Published:** 2020-10-08

**Authors:** Leslie Raphael de Moura Ferraz, Alinne Élida Gonçalves Alves Tabosa, Débora Dolores Souza da Silva Nascimento, Aline Silva Ferreira, Victor de Albuquerque Wanderley Sales, José Yago Rodrigues Silva, Severino Alves Júnior, Larissa Araújo Rolim, Jorge José de Souza Pereira, Pedro José Rolim-Neto

**Affiliations:** 1 Laboratório de Tecnologia Dos Medicamentos, Department of Pharmaceutical Sciences, Federal University of Pernambuco, Av. Prof. Arthur de Sá, s/n, Cidade Universitária, Recife, PE 50740-521 Brazil; 2 Laboratório de Terras Raras, Departamento de Química Fundamental, Federal University of Pernambuco, Av. Jornalista Aníbal Fernandes, s/n - Cidade Universitária, Recife, PE 50740-560 Brazil; 3 Central de Análise de Fármacos, Medicamentos e Alimentos, Federal University of Vale Do São Francisco, Av. José de Sá Maniçoba, s/n, Centro, Petrolina, PE 56304-917 Brazil; 4 Laboratory of Immunopathology Keizo Asami (LIKA), Federal University of Pernambuco, Recife, Brazil

**Keywords:** Infectious diseases, Parasitic infection, Drug development, Organic-inorganic nanostructures, Characterization and analytical techniques, Design, synthesis and processing, Drug delivery

## Abstract

Chagas disease (CD), caused by the flagellate protozoan *Trypanosoma cruzi*, is one of the major public health problems in developing countries. Benznidazole (BNZ) is the only drug available for CD treatment in most countries, however, it presents high toxicity and low bioavailability. To address these problems this study used Zeolitic Imidazolate Framework-8 (ZIF-8), which has garnered considerable attention due to its potential applications, enabling the controlled delivery of drugs. The present work developed and characterized a BNZ@ZIF-8 system, and the modulation of BNZ release from the ZIF-8 framework was evaluated through the in vitro dialysis release method under sink conditions at different pH values. Moreover, the in vitro evaluation of cell viability and cytotoxicity by MTT assay were also performed. The dissolution studies corroborated that a pH sensitive Drug Delivery System capable of vectorizing the release of BNZ was developed, may leading to the improvement in the bioavailability of BNZ. The MTT assay showed that no statistically significant toxic effects occurred in the developed system, nor significant effects on cell viability.

## Introduction

Chagas disease or American trypanosomiasis is a neglected tropical disease caused by the flagellated protozoan *Trypanosoma cruzi*. Although it is endemic in about 21 developing countries in Latin America, it is a public health problem on other continents due to the intense immigration movement of the infected population. It is estimated that, globally, 6 to 7 million people are infected with *T. cruzi* whose contamination occurs through contact with the waste of infected blood-sucking triatomine bugs or by blood transfusion, work accidents and maternal transmission^1–3^.

*T. cruzi* infection is potentially fatal. It induces distinct clinical aspects, presenting an acute phase that might be asymptomatic and last an average of 2 months, being succeeded by a chronic phase which is more severe and typically extends throughout the life of the host. Its clinical manifestations are characterized by the Romaña sign or the skin lesion called “chagoma”, followed by fever, myocarditis, electrocardiographic changes, lymphadenopathy and hepatosplenomegaly; in the most severe cases. Currently, chemotherapy treatment uses Benznidazole (BNZ) as the first choice. BNZ, chemically *N*-benzyl-2-(2-nitro-1H-imidazol-1-yl)acetamide (C_12_H_12_N_4_O_3_—260.25 g mol^−1^), is the only drug available for treatment of Chagas disease in countries such as Brazil, Argentina, Chile and Uruguay and the only one marketed for this purpose in Latin America^2–5^.

The recommended treatment is 5 to 7 and 5 to 10 mg kg^−1^ orally, divided into two or three daily doses for 60 days for children and adults, respectively. First, BNZ was formulated only as tablets for oral administration (100 mg), culminating in treatment limitations mainly in child patients, where the drug was grossly fragmented, increasing the risk of incorrect doses and the incidence of toxic effects or therapeutic failure. Then, in 2011, a pediatric dose of BNZ (12.5 mg) tablets began to be marketed, the result of an initiative by the Drugs for Neglected Diseases Initiative and Brazilian LAFEPE, the only producer of the drug. Nowadays, efforts are being made to develop more effective dosage forms, capable of increasing patient compliance and reduced toxic effects^4–6^.

Although BNZ has been used since the 1970s, its mechanism of action is not well understood. Several observations in the literature converge to formation of toxic metabolites to the parasite’s DNA and RNA, proteins and lipid molecules by reducing the nitro group of the drug mediated by *T. cruzi* type I nitroreductase. Effective against epimastigotes, trypomastigotes and amastigotes, these studies suggest that BNZ increases the concentration of superoxide anions and nucleophilic metabolites at the mitochondrial level, meanwhile forms covalent bond of its nitro group with *T. cruzi* macromolecules reducing the parasite’s metabolism. Other findings also suggest that BNZ increases the phagocytosis and lysis of *T. cruzi* through an interferon-gamma-dependent mechanism (INF-g) and inhibits NADH-fumarate reductase enzyme and therefore the growth of the parasite^7–9^.

Furthermore, according to the biopharmaceutical classification system, BNZ is classified as class II, thus, it has low solubility in aqueous fluids (about 0.2 mg mL^−1^). Consequently, it has limited absorption due to the low dissolution rate. In addition, its low bioavailability requires a considerable dose of drug, which may be related with its high toxicity, leading to some serious adverse reactions, such as skin allergies, bone marrow suppression and peripheral neuropathy. Nevertheless, it is still used in the acute phase, with chances of cure, and in the chronic phase, preventing the disease progression. Therefore, as it is a worldwide public health problem, there is a certain urgency for the development of therapeutic alternatives^6,10–12^.

Due to the low solubility in aqueous medium, BNZ has a reduced absorption in the gastrointestinal tract, and therefore, has low bioavailability. Thus, to address these problems, increasing solubility and modulating drug release through smart excipients the Drug Delivery System (DDS) has been developed (Gomes et al.^13^; Lima et al.^14^; Soares-Sobrinho et al.^15^). Among the most recent DDS the Metal Organic Frameworks (MOFs) have received growing attention. MOFs are organic–inorganic hybrid materials that have promising properties to be used as carriers of drugs, such as: improving bioavailability and biocompatibility of drugs, large surface area, high thermal stability, insertion capacity of ionic compounds and small molecules, and possibility of promoting controlled release of substances^13–20^.

Zeolitic Imidazolate Framework (ZIF) is a subclass of MOF composed of tetrahedral coordination metals, usually zinc or cobalt, interconnected through imidazoles or imidazolates linkers. Although they are similar to zeolites, ZIFs are getting more attention due to a larger surface area, permanent porosity, different topologies and high thermal stability. Among the ZIFs, ZIF-8 has recently been used for several purposes, such as: membrane constituents, gas exchange and storage, biosensors, catalysis agents and drug carriers for the development of DDS^20–24^.

The ZIF-8 has tetrahedral zinc as a coordination metal and is stabilized by the bridges formed by 2-methylimidazole at an angle of 145°, just like sodalites. It presents itself as a lamellar structure maintained through compensation between π–π stacking interaction and hydrogen bonds, resulting in a one-dimensional polymeric chain of densely interconnected lamellae. This conformation gives the coordination framework a central octahedral pattern shared by tetragonal and hexagonal faces, with cavities of about 11.6 Å and 0.4 nm^3^ that contribute to a wide surface area and that can be used for the delivery of drugs and the use of the ZIF-8 as a carrier^25–30^.

Because it is the second most abundant metal in the human body and the imidazole group is found in the amino acid histidine, ZIF-8 has good biocompatibility and safety^26^. Thus, ZIF-8 has potential as a pharmaceutical adjuvant due to its adsorption capacity followed by the release of drugs, whether hydrophilic or hydrophobic given its organic–inorganic composition^28–30^. In addition, as it has a flexible structure, the ZIF-8 presents a phenomenon called breathing, which ends up promoting a modulated release of drugs. The molecule has a simple and fast synthesis and is still highly chemically and thermally stable, withstanding temperatures of up to 400 °C before decomposition, which allows a series of industrial operations^31,32^. The development of DDS using ZIF-8 as smart excipient might be an important tool for the transport and control of drug release kinetics from a pharmaceutical dosage form with the main objective of establishing constant therapeutic plasma levels, within the therapeutic index, with high bioavailability and less toxic effects^33–36^.

Therefore, this study aimed to analyze the release kinetics and cell viability of a new drug delivery system based on BNZ carried by the ZIF-8 framework, using in vitro dialysis and cytotoxicity tests in order to provide the necessary subsidies for the development of new pharmaceutical products for Chagas disease.

## Results and discussion

### Evaluation of drug incorporation in ZIF-8 system

**Figure 1:**
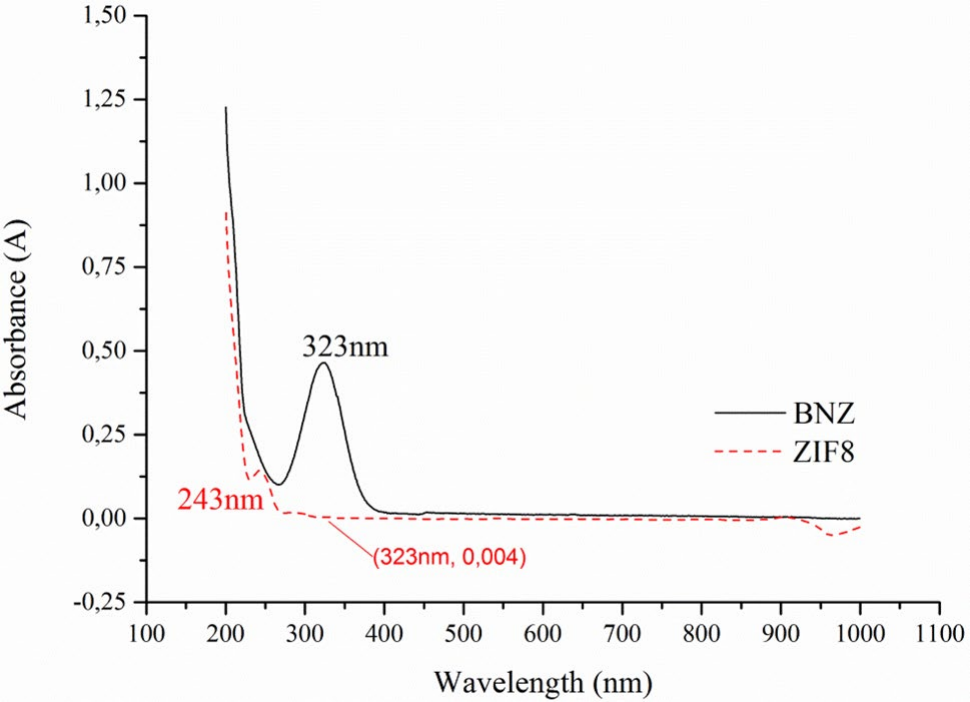
UV–Vis spectra of BNZ and ZIF-8.

Through absorption spectroscopy in the UV–Vis region it was possible to determine the reading wavelength to be used for the quantification of BNZ incorporation. Based on the scan (200–1000 nm) (Fig. 1), the BNZ solution (15 μg mL^−1^) had maximum absorption at 323 nm, while the ZIF-8 at 243 nm. In order to prove that ZIF-8 would not be a contaminant capable of interfering with the selectivity of the BNZ quantification method, a quantity of 10 mg of ZIF-8 was suspended in 100 mL of ultra-pure water, followed by filtration (pore filter: 0.22 μm) and quantification using UV–Vis.

Although ZIF-8 also shows peak absorption in the UV–Vis region, the method did not present selectivity problems because the material does not absorb at the same wavelength. In addition, ZIF-8 is insoluble in water and in nonpolar solvents, being excluded from the analyte the moment the sample is filtered^26^.

Thus, it was possible to build daily calibration curves, based on the method developed by Soares-Sobrinho et al.^15^ making it possible to calculate the apparent incorporation of BNZ into the ZIF-8 network. The experiment aimed to analyze the drop in drug concentration, justified by its incorporation into the ZIF-8 network. This decrease was evaluated daily and the percentage of incorporated BNZ was measured by subtracting the value of the actual concentration used in the experiment by the dissolved concentration found, represented in terms of percentage, as described by Eq. 1 in experimental section. From the incorporation curves it was possible to observe that, after 4 days of intermittent agitation, the value of IE% was 38%.

### Thermal analysis (DSC, TG/DTG)

**Figure 2:**
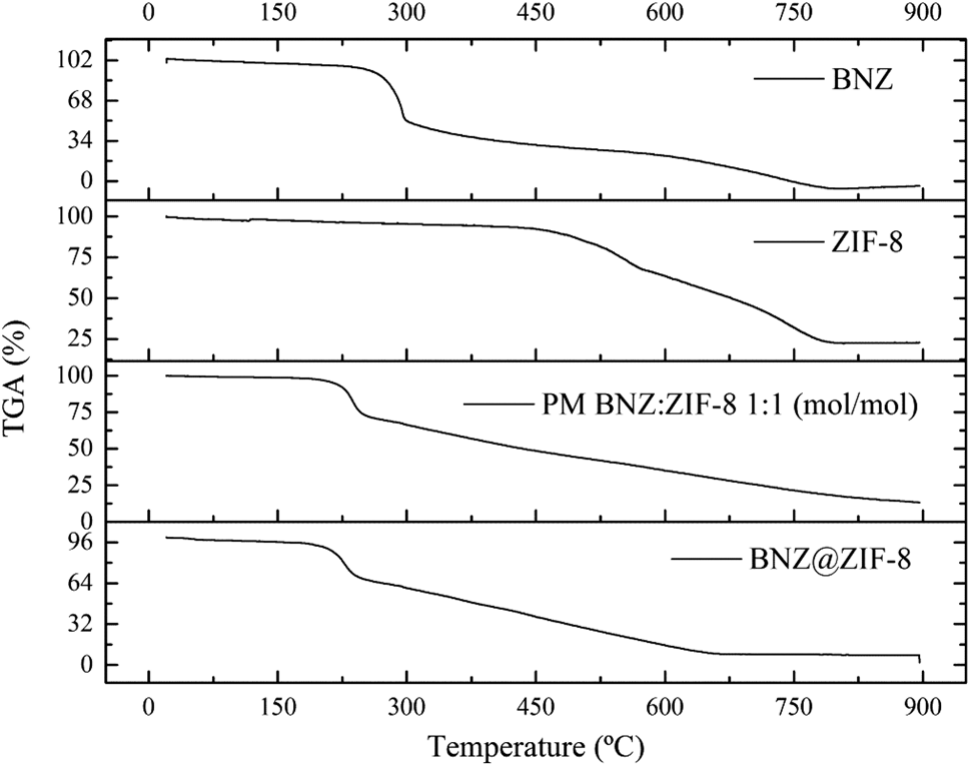
TG curves of BNZ, ZIF-8, physical mixture (PM) and BNZ@ZIF-8 (β = 10 °C min^−1^).

BNZ TG curve (Fig. 2) showed a mass loss of 1.62%, between 30 and 105 °C relative to the water content in the sample. Thermal degradation of the isolated drug was observed in only one single event (257.15–297.13) (DTG peak = 287.43 °C). A mass loss of 45.66% was found.

In the TG curve of ZIF-8 (Fig. 2), the high thermal stability of the molecule was observed^31,32^. First, it was possible to show the mass loss of up to 2.16% in the temperature range between 30 and 125 °C. This value may be related to the loss of water molecules in cavities or on the surface of ZIF-8^31^. At 460.40 °C, there were loss of only 7% of the initial mass, which evidences the high thermal stability of the ZIF-8. This value can also indicate the exit of water molecules connected to the ZIF-8 network. In the range of 535.69–578.16 °C it was possible to observe a significant mass loss—better evidenced by the DTG curve—of 24.32%, related to the molecule organic portion degradation, the imidazolate. Since then, the sample presents slow decay, with low resolution, and no apparent peaks, being difficult to identify them even by the DTG curve, probably caused by thermal decomposition of the inorganic portion of ZIF-8, until the formation of zinc oxide (620.35–720.11 °C—41.91% mass loss). These results are in accordance with previous studies^37–39^.

In the TG curve of physical mixture (PM) (Fig. 2) it was possible to see the anticipation of the thermal degradation of the isolated drug, now occurring in the range of 229.89–247.23 °C (DTGpeak = 238.04 °C), subsequent to drug melt. However, a significant reduction of mass loss (24.49%) was observed, suggesting a certain thermal protection in the reduction of mass loss. In relation to ZIF-8 degradation, the first event of thermal degradation—related to degradation of imidazolate—was observed in a range quite different from that found for ZIF-8 alone (between 410.89 and 448.54 °C), also presenting reduction of mass loss (11.55%). This fact suggests that the physical interaction between drug and ZIF-8 may destabilize the organic portion of the latter. Then, the TG curve decayed to approximately 975.07 °C corresponding to residual zinc oxide (36.78% of mass loss).

In the analysis of the BNZ@ZIF-8 (Fig. 2), a new mass loss event was observed, between 50.13 and 87.94 °C, with a mass loss of 1.97%. Probably, this event is related to the volatilization of acetone that still remains inside the ZIF-8 network, since the decay can be observed from room temperature, with acetone having low vapor pressure and boiling point. This value, smaller than that found for the isolated ZIF-8, still shows that there is a smaller amount of water (moisture) present in the cavity of the ZIF-8, and therefore, gives space for the connection with the molecules of the drug. The rest of the TG curve showed some consonance with the previous results, from the aqueous medium obtained system. An anticipation of the thermal degradation of the drug over the isolated BNZ (223.39–245.34 °C) was also observed^23,26,40^.

However, there was an even more significant reduction in mass loss (26.49%). The degradation of the inorganic portion of ZIF-8 occurred in the range of 321.02–434.88 °C, with a mass loss of 16.15%; followed by thermal decomposition to zinc oxide (568.51–613.31 °C and 32.78% mass loss). Although it has been shown that ZIF-8 degradation values were anticipated, both showed a significant reduction of the decomposed content. The difference between this thermal profile and that evidenced by PM may be a great indication of the actual formation of systems, corroborating the fact that the drug is actually present in the ZIF-8 cavities^23,40^. However, new investigations about this fact should be made in order to analyze what, in fact, promoted this behavior.

**Figure 3:**
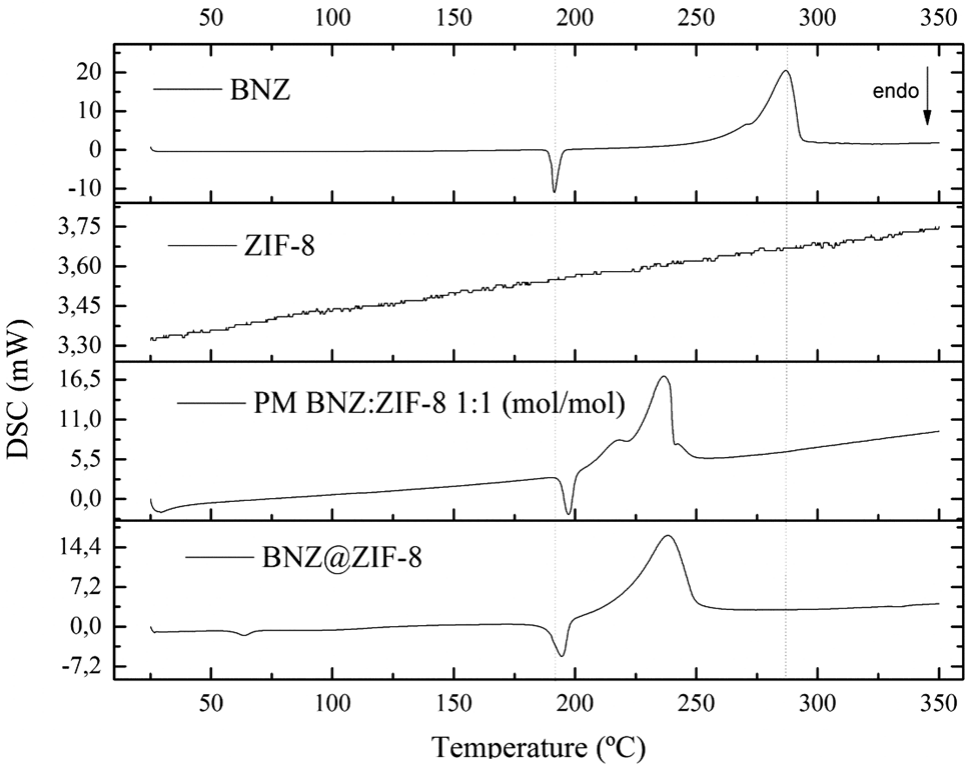
DSC curves of BNZ, ZIF-8, physical mixture (PM) and BNZ@ZIF-8 (β = 10 °C min^−1^).

BNZ DSC curve (Fig. 3) showed an intense and defined endothermic peak in the temperature range between 190.04 and 194.36 °C (Tpeak = 191.44 °C) (ΔH = 195, 52 mJ). Then, the exothermic peak related to drug degradation was observed in the range of 272.57–292.79 °C (Tpeak = 286.90 °C), which showed large energy release (ΔH = 1.8 J). Similar results were described by Santos et al.^41^

**Figure 4:**
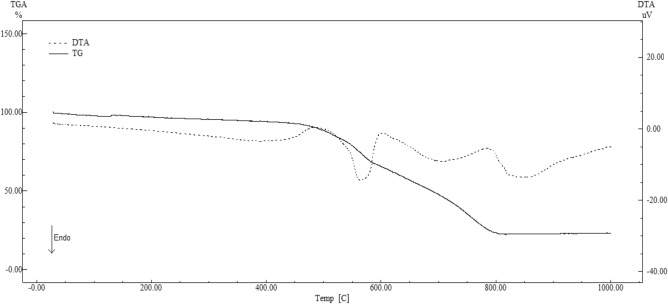
DTA curve of ZIF-8 (β = 10 °C min^−1^).

The DSC curve of ZIF-8 (Fig. 3) demonstrates the absence of peaks in the temperature range used, a feature inherent in the nature of the molecule. Since it is an organic–inorganic hybrid molecule, the ZIF-8 degradation events were analyzed through the DTA curve (Fig. 4), which enabled the sample to heat up to 900 °C. In that point, it was possible to observe two endothermic events regarding phase transition: the first one between 535.69 and 578.16 °C; and the second, between 620.35 and 720.11 °C. These values and their mass losses are described in the discussion of the TG curve of ZIF-8. Such behavior can be observed in zinc-containing materials. Similar results were discussed by Blachnik and Siethoff^42^. Such solid–solid phase transitions suggest conformational changes of the alkyl chains, where the number and size of the transformations occur as a function of the length and number of the alkyl chains^42^.

From the DSC curve of the PM (Fig. 3) it was possible to evidence the anticipation of the drug fusion event (187.82–194.28 °C). Although this fact can identify interactions between the components of a formulation, the curve also showed the reduction of enthalpy energy related to the event (ΔH = 195.52 mJ). This phenomenon is characteristic of polymeric materials, such as: PEG, PVP, HPMC; which makes it possible to increase the solubility of the material, since there is need for a smaller amount of energy to solubilize the drug. Such behavior has already been widely discussed by previous studies of the group^43,44^.

Thus, it is clear that simple physical mixing promotes the formation of a drug–excipient interaction, even without causing the formation of the system itself. On the other hand, there was an intense anticipation of the event related to the degradation of the drug (Tpeak = 231.08 °C), almost occurring subsequent to the fusion of the same. However, there was lower energy release (ΔH = 1.09 J). These values are in line with those evidenced by the DTA curve.

BNZ@ZIF-8 DSC curve (Fig. 3) shows an endothermic event between 54.17 and 62.85 °C, regarding the volatilization of acetone. This behavior, different from that presented in PM, can characterize the formation of the BNZ@ZIF-8. A much more discrete anticipation (184.44–192.91 °C) (Tpeak = 189.97 °C) was observed in the drug fusion. This variation—approximately 2%—is described by many authors as an acceptable range of compatibility between components of the same formulation^43,45^. Therefore, this behavior can identify that the system obtained in acetone is, in fact, more efficient for drug incorporation. However, the value for the enthalpy change was higher in comparison to the isolated drug (ΔH = 261.25 mJ), which may indicate that the system was formed, since a new thermal profile was evidenced. Regarding drug degradation, the exothermic event was anticipated (216.72–244.86 °C) (Tpeak = 234.14 °C), releasing an energy of 1.8 J, identical to that presented by the isolated drug.

All the above-mentioned information shows the importance of thermal analysis as a technique for the characterization of DDS. Through the TG/DTG, DTA and DSC curves it was possible to corroborate the fact that the acetone system made possible a more efficient incorporation of the drug, either by changing the thermal profile or by the compatibility between the used components.

### X-ray diffraction spectroscopy

It was possible to identify the characteristic peaks of both BNZ crystals (7.36°, 16.28° and 21.86°) and ZIF-8 (7.44°, 10.46° and 12.78°, corresponding to reflections 011, 002 and 112, relating to a body-centered hub). These data were used to calculate the basal spacing in order to establish a reference value to characterize the insertion of the BNZ molecule in ZIF-8. These results are in line with recent works^12,31,46^.

The BNZ@ZIF-8 system also presented crystalline behavior, resulting in the sum of the XRD profiles of the isolated substances. However, different from PM, the system showed the most characteristic peaks of the BNZ (7.36°, 10.88°, 16.82° and 21.88°), which suggests the formation of the coupled system, even though it is only characterized by the physical adsorption of the BNZ on the surface of ZIF-8.

**Figure 5:**
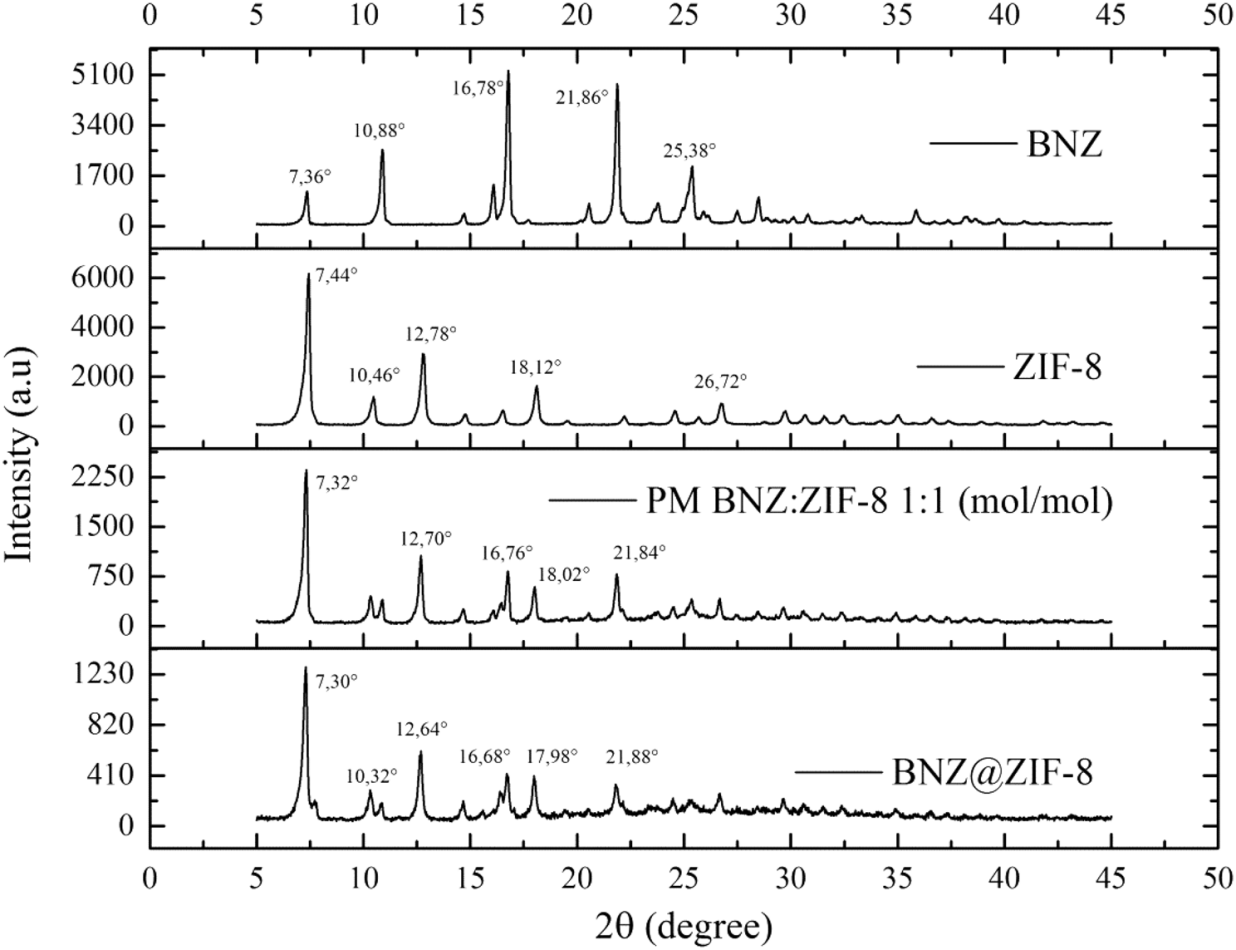
XRD of the BNZ, ZIF-8, PM and the BNZ@ZIF-8 with their respective main peaks.

In this material, a reduction of the peak intensity characteristic of ZIF-8 (7.44°) was observed indicating the strong attraction between the BNZ and the ZIF-8 network. Similar results were evidenced by Liédana et al.^20^ It is important to notice that the obtained XRDs demonstrate that the structural integrity of ZIF-8 remains unchanged after the adsorption of the BNZ. This is of great importance because it shows that even though the formation of the systems promotes some reduction of the peaks of the molecule, and the crystalline structural integrity of the ZIF-8 is maintained^34–36,40^. The XRD results of all materials are summarized in Fig. 5.

Basal spacing values, defined by the Bragg equation, were used as a parameter to confirm the insertion of the drug into the ZIF-8 molecule. Values greater than baseline suggest that the drug was inserted into the molecule and, therefore, dilates the space between the lamellae. However, the discrete increase in basal spacing was observed when the isolated ZIF-8 was compared to the PM and to the obtained system (Table 1), mainly at the peak of reflection 110, the most characteristic of ZIF-8 crystallinity and of the rhombic dodecahedral of its crystals (He et al.^41^). This fact suggests the formation of the system but suggests that it is the product of an adsorption of the drug to the surface of the ZIF-8, characterizing a physical interaction, since the increase of the basal spacing is very small.

**Table 1:** Calculation of the basal spacing of the reflection peaks 110, 002 and 112 of ZIF-8, Physical Mixture and Systems obtained in water and acetone.

Sample	Peak (° 2θ)	Reflection	Basal spacing (Å)
ZIF-8	7.44	110	11.87
	10.46	002	8.71
	12.78	112	6.94
PM	7.32	110	12.04
	10.4	002	8.51
	12.70	112	6.97
BNZ@ZIF-8	7.3	110	12.11
	10.32	002	8.10
	12.7	112	6.97

### Fourier transform infrared absorption spectroscopy

**Figure 6:**
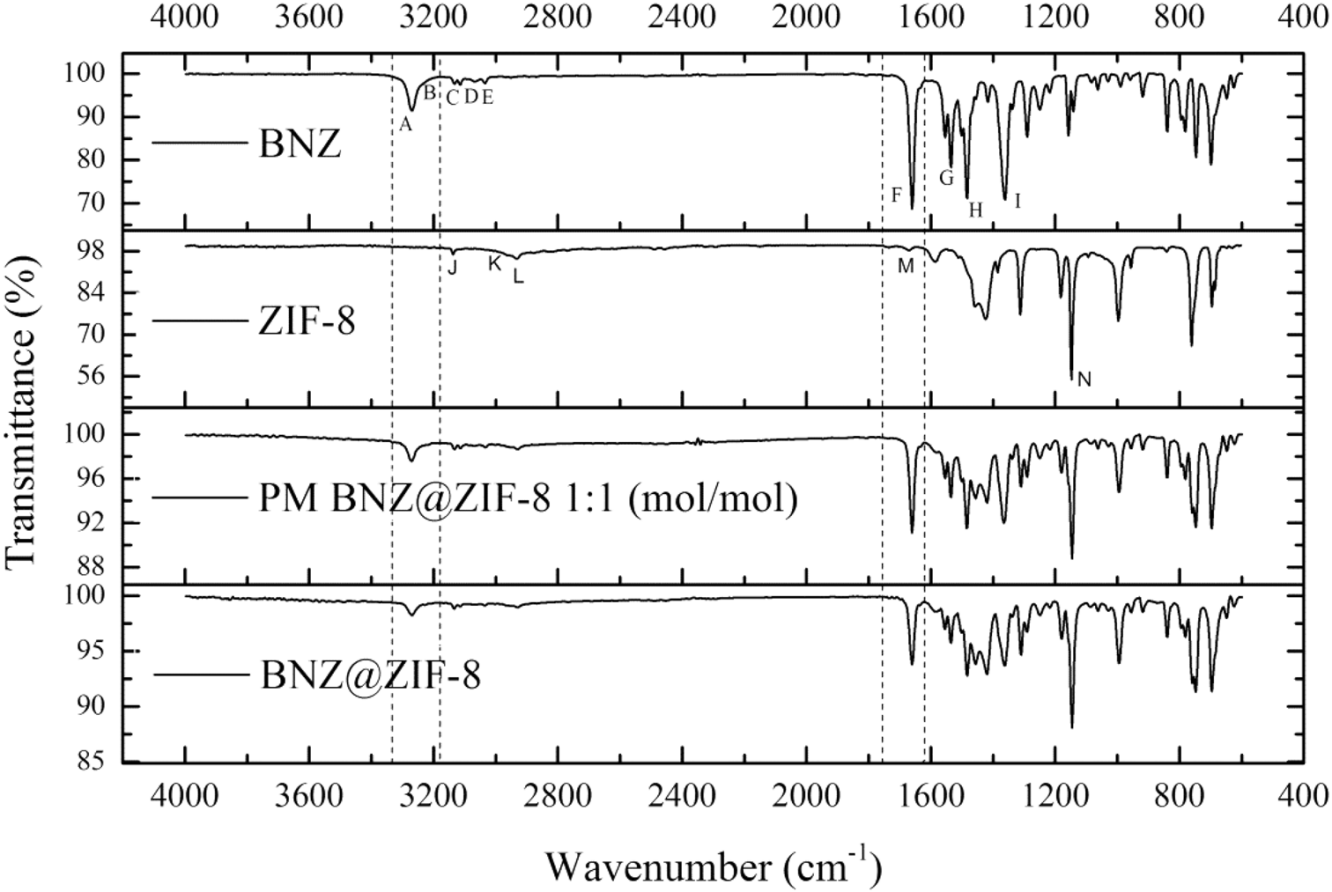
Infrared spectra of BNZ, ZIF-8, physical mixture (PM) and BNZ@ZIF-8.

The BNZ spectrum (Fig. 6) shows characteristic peaks, especially when considering typical bands of amides (N–H stretching vibration), carbonyl stretching (amide band I) and N–H deformation (amide band II), in addition to vibrations resulting from the benzyl and imidazole groups, and the nitro group. The band from the N–H stretching vibrations is located at 3266 cm^−1^, the carbonyl stretching band at 1664 cm^−1^ and the NH (amide II) deformation at 1552 cm^−1^, characterizing the secondary amide. In addition, the band at 1292 cm^−1^ is attributed to the C–N stretch. The set of bands at 3033, 3068, 3112 and 3269 cm^−1^ arise from the symmetrical and asymmetric stretching vibrations of the benzene group and the stretching of the aromatic C–H, as shown in Fig. 6.

The region of the harmonics and combination bands (2000 to 1667 cm^−1^), often useful in determining the number and position of substituents on aromatic rings, appears to be less informative in the spectrum, not being observed the four small bands that would characterize mono-substituted benzene present in the molecule. The band at 1355 cm^−1^ refers to vibration of the symmetrical stretching of the nitro group^44^.

Looking at Fig. 6 referring to ZIF-8, at 3132, 2962 and 2936 cm^−1^, the presence of C–H aromatic compounds, asymmetric axial deformation and aliphatic C–H stretching relative to the imidazole ring, respectively. In 1670 cm^−1^ a stretch band of C=C was observed, while the absorption C–N bands appeared at 1100 to 1400 cm^−1^ region (Park et al.^31^). It is not possible to observe the behavior of the Zn–N stretch at 450 and 400 cm^−1^, considering that the analyzes were done in equipment that operated up to 600 cm^−1^.

The PM spectrum (Fig. 6) corresponds to the overlapping of the same bands of BNZ and ZIF-8 when these are analyzed in isolation, it is possible to note the presence of their characteristic peaks. This result suggests a physical interaction only between the drug and ZIF-8 due to the sum of the profiles of the isolated materials.

Analyzing the BNZ@ZIF-8 (Fig. 6), the peaks of the isolated BNZ and ZIF-8 are not well evidenced due to their overlapping. However, it was possible to suggest the presence of the drug in question, due to the presence, in 3266 cm^−1^ and 1292 cm^−1^, of the axial deformation of the N–H and C–N bond, respectively. In 1664 cm^−1^ there is a decrease in the intensity in the carbonyl band, at 1355 cm^−1^ for the nitro group and 1552 cm^−1^ corresponding to the secondary amide. In addition, there was a decrease in the intensity of the absorption band related to the C–N group of ZIF-8 (1400 and 1302 cm^−1^), confirming that it interacts effectively with BNZ^26^.

Thus, when comparing the infrared spectra of BNZ@ZIF-8, PM and the isolated substances, it was observed that the system presented the main peaks in a lower intensity, indicating, therefore, the formation of the system due to the signs of interaction between BNZ and ZIF-8^40^.

### In vitro dialysis release method and the breathing phenomena

The in vitro release assay through a dialysis membrane was performed in order to estimate the BNZ permeation from the ZIF-8 network. The diffusion of the drug through the dialysis membrane pores (1000 Da) is possible due to its molecular size (about 260.25 Da). Thus, the BNZ molecules are able to be released through the dialysis membrane while the ZIF-8 network is dissociated or eroded. In contrast, ZIF-8 cannot permeate through the dialysis membrane due to its large structural size and rigid polymeric matrix.

**Figure 7:**
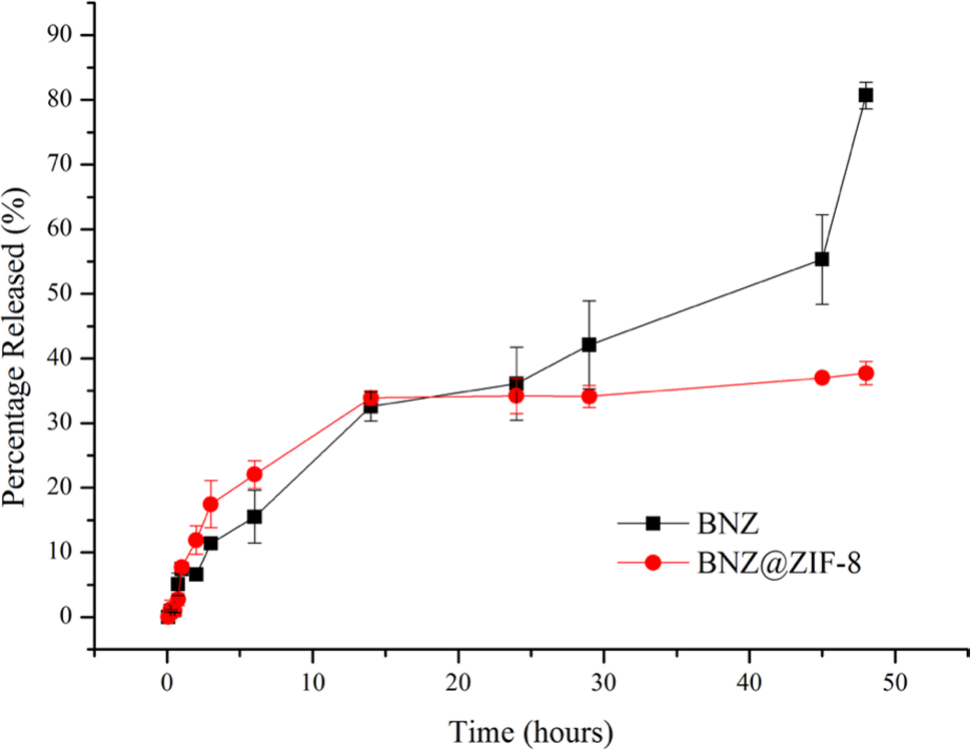
In vitro cumulative drug release (%) of BNZ and BNZ@ZIF-8 system through dialysis membrane (10,000 Da) under sink conditions at pH 4.5.

At pH 4.5 (Fig. 7), the BNZ alone achieved 80% of release after 48 h with an almost linear release kinetics. On the other hand, the BNZ@ZIF-8 system, in the same time interval, presented a slower release of BNZ (34%), without oscillations. After 25 h, the drug and the BNZ@ZIF-8 system exhibit a similar release, approximately 39 and 35%, respectively. However, after this point the system presents the formation of a *plateau*, stabilizing the release of the drug in this same range until the end of the experiment, while the drug alone continues to be released in a linear kinetics, reaching the percentage expected for the end of the test.

In order to compare the dissolution profiles between the BNZ and the system, the model-independent method that calculates the value of ƒ2 was used. The value of 190.87 shows that the release profile among them is different, once ƒ2 > 100.

**Figure 8:**
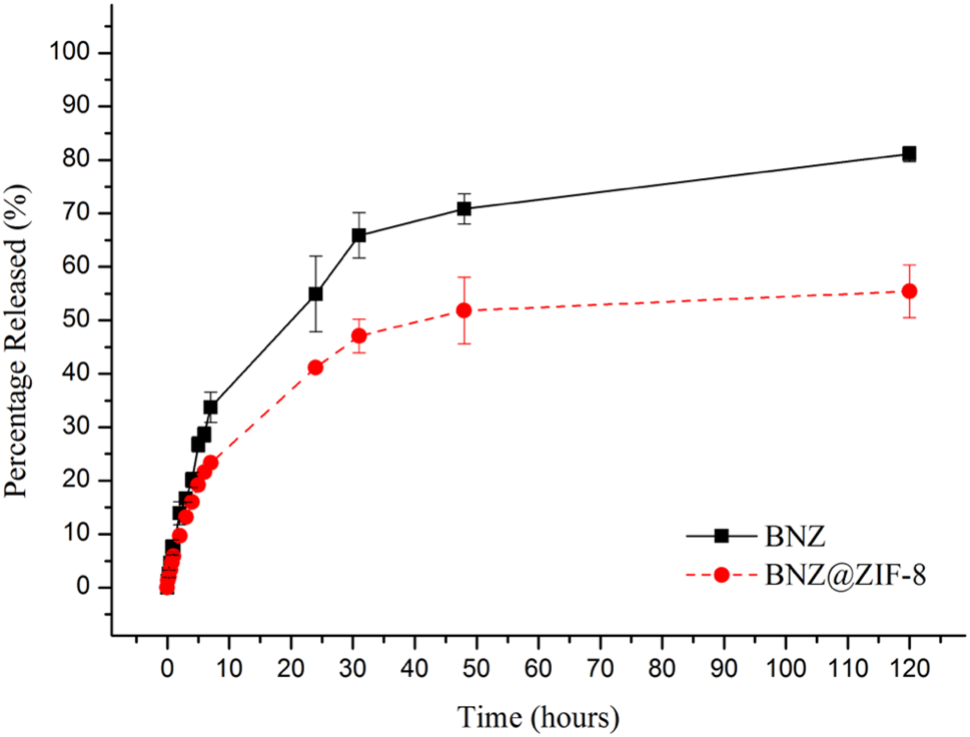
In vitro cumulative drug release (%) of BNZ and BNZ@ZIF-8 system through dialysis membrane (10,000 Da) under sink conditions at pH 7.6.

At pH 7.6 (Fig. 8), the isolated BNZ achieved 80% of release after 120 h of assay, due to its low aqueous solubility. In contrast, the BNZ@ZIF-8 system presented a slower release, excluding the burst effect presented by the BNZ alone. After 7 h, the BNZ alone achieved 40% of release, while the BNZ@ZIF-8 system released only 23% of the drug. This difference of percentage—approximately 20%—remains constant up to 120 h, where the system reaches only 57% of drug release.

It was evident that, in fact, the drug release modulation occurred, since the release profiles were not similar due to the obtained ƒ2 value: 213.06 (> 100).

**Figure 9:**
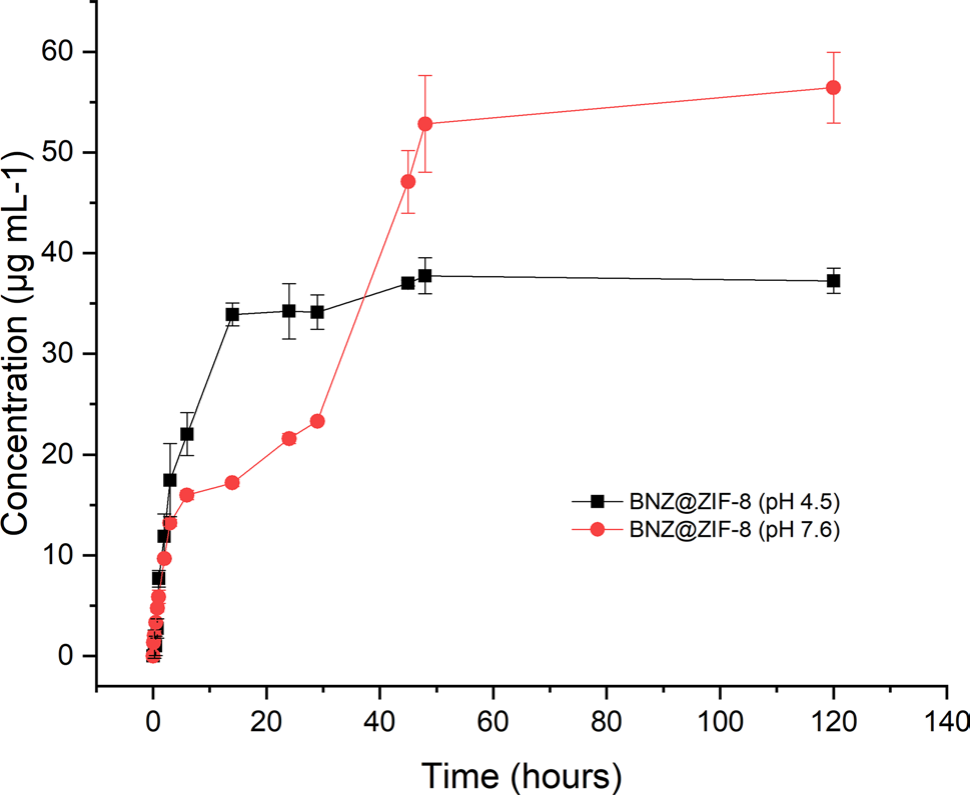
Comparative profile of the BNZ@ZIF-8 system through dialysis membrane (10,000 Da) under sink conditions at pH 4.5 and 7.6.

Figure 9 provides a comparison between the results obtained from dialysis in sink conditions at different pH values. It was possible to observe a fast release at pH 4.5, with a pronounced and almost linear burst effect, releasing about 34% in 14 h. Afterwards, a *plateau* concentration maintenance is observed, reaching approximately 38% of release until the end of the experiment. MOFs usually show the structural transition phenomena upon the input of external stimuli, known as the breathing phenomenon. This phenomenon is common among MOFs. Due to the flexibility of these molecules in an acidic environment, they open exit gates for the adsorbed substances that were previously trapped by the coordination polymer^33,46^.

Also called “easing”, the breathing phenomenon of ZIF-8 is extremely important in drug delivery applications, allowing the adsorption and diffusion of large molecules. The breathing flexing of the ZIF-8 framework is the process in which the movement/twisting of the imidazolate linkers in the network, providing a greater opening of the crystal pore windows, causing a dilation or contraction of the pores, directly interfering with the adsorbed load and in the diffusion of molecules. ZIF-8 presents the beginning of the dissociation of the coordination network in the pH range between 5.0 and 6.0, and intense erosion in an even more acidic pH, which can promote the network’s “breathing”, opening of spaces between the polymeric mesh and, consequently, intensifying the drug release which, in this case, can be modulated by changing the pH^37,38,47^.

At pH 7.6, an attenuation of the burst effect is observed, since only 17% of BNZ is released in the same time interval of 14 h. The release follows slowly, modulated, with about 53% released in 48 h, where a balance is established, since the release is about 56% until 120 h. Then, the dissolution studies corroborated that a pH sensitive DDS capable of vectorizing the release of BNZ was developed.

It is evident that a lower percentage of drug is released from the BNZ@ZIF-8 system in a time interval, comparing to the BNZ alone. This behavior should be explained by the interactions between the drug that is closely linked to the ZIF-8 coordination network, although only physically adsorbed. It should be noted that, while the drug dissociates and diffuses, a new sorption and desorption equilibrium of the drug can be achieved, which further prolongs even more, the release time interval.

An important factor to be considered during dialysis is that the membrane acts as a barrier between ZIF-8 suspension after drug release and the external environment. However, to be quantified in the external environment, the drug must diffuse not only through the ZIF-8 network, but also through the dialysis membrane.

The pH values used in this study are extremely interesting for the pharmaceutical field in the development of pH-dependent DDS. For example, by modulating the release of the drug to occur at an acidic pH only, it is possible to avoid an intense drug release in bloodstream and thus, reducing the incidence of adverse effects and increasing the selectivity of the treatment. This fact is particularly interesting for the treatment of Chagas disease.

In the life cycle of *T. cruzi*, the parasite provides mechanisms of action capable of favoring its entry into the host-cell cytosol. For example, the rupture of the parasites phagocytic vacuole marks the transformation of the trypomastigote phase into amastigote. At this stage, about 70% of the parasites will be free in the cytosol 2 h after infection. Some studies suggest that this is due to the secretion of acidic substances, such as hemolysin, which has maximum activity at pH 5.5. Some of this studies showed that the rupture of the vacuole occurs around pH 6.0 and, with the addition of slightly more basic substances (pH 6.2), the escape of parasites is inhibited. Therefore, it is inferred that the release of the parasites and the continuity of the life cycle of the parasite are related to a change in pH to slightly more acidic levels^39,48,49^.

### In vitro evaluation of cell viability and cytotoxicity using the MTT assay

**Figure 10:**
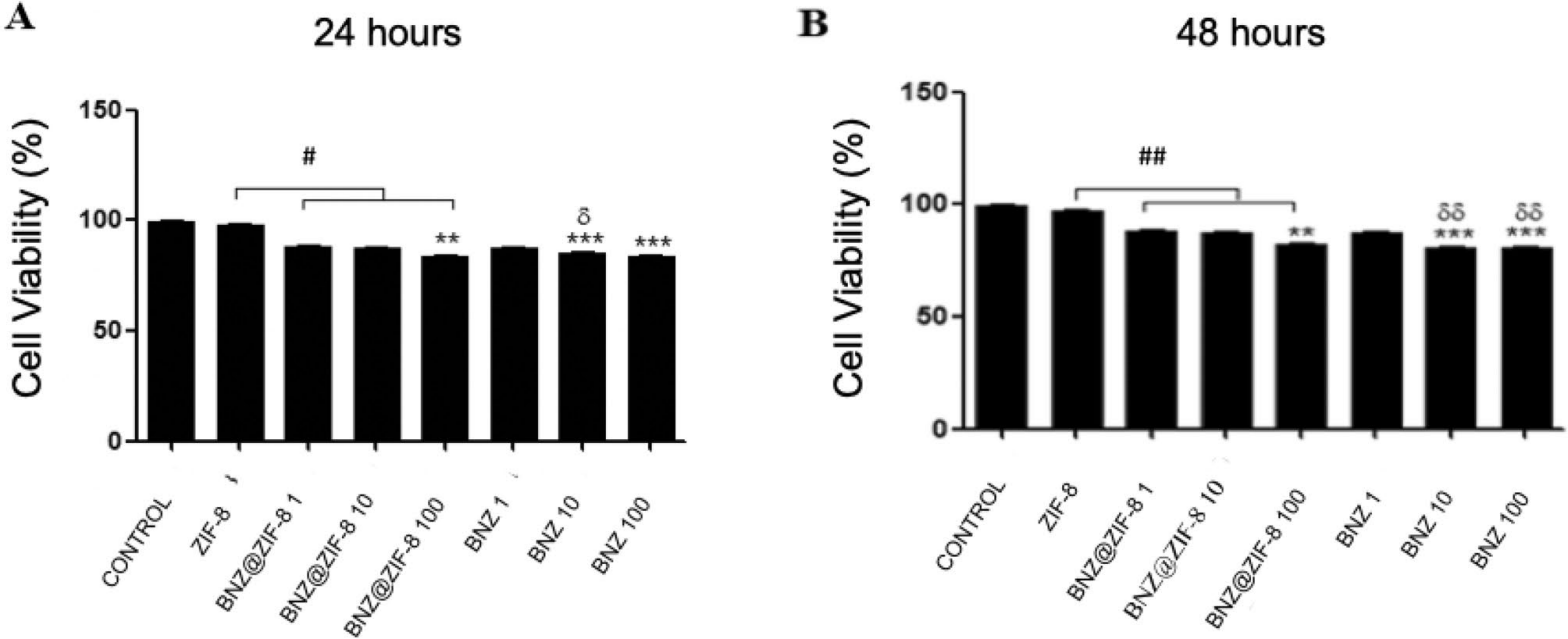
Effects of BNZ, ZIF-8 and BNZ@ZIF-8 at different concentrations on human cell viability (PBMC) according to the MTT assay at exposure time: (**A**) 24 h and (**B**) 48 h (**p < 0.05, ***p < 0.001 are related to significant differences between the tested and control groups; #*p* < 0.05 to the comparison between activity of ZIF-8 alone and when combined in the systems and δ*p* < 0.05 the difference between the system and the drug alone, both at 10 μg/mL).

The low cytotoxic activity of the materials was verified. Even with the highest concentrations tested (100 μg mL^−1^), all samples induced low levels of cell death (viability > 80%) over a 24-h period (Fig. 10A). After 24 h, ZIF-8 (150 μg mL^−1^) showed low cytotoxic potential (viability of 98.2% *vs* control 99.6%). The BNZ showed viability varying between 83.9 and 87.6%. For BNZ@ZIF-8 systems, cell viability values were 88.6%, 87.7% and 84%, for the doses of 1, 10 and 100 μg mL^−1^, respectively. There were significant differences between the tested and control groups (**p < 0.05, ***p < 0.001), and a significant difference between the cytotoxic activity of ZIF-8 alone and when combined in the systems (#*p* < 0.05). Furthermore, there was a difference between the system at 10 μg mL^−1^ when compared to the drug alone at the same dosage (δ*p* < 0.05).

After 48 h of exposure (Fig. 10B), the viability of the ZIF-8 exposed cells alone remained high (97.3%) versus a range of 80 to 87% of the BNZ groups. In the BNZ@ZIF-8 system, the values were 88.5%, 87.7% and 82.4%, for 1, 10 and 100 μg mL^−1^, respectively. There were significant differences between the tested and control groups (**p < 0.05, ***p < 0.001), and a significant difference between the ZIF-8 alone activity when compared to the BNZ@ZIF-8 systems (*p* < 0.05). Moreover, it was possible to observe a difference between the BNZ@ZIF-8 system at 10 μg mL^−1^ and the BNZ alone at the same concentration, as well as the system BNZ@ZIF-8 activity (100 μg mL^−1^) when compared to the BNZ alone at this same concentration (δδ*p* < 0.05). The slow release of the drug by the BNZ@ZIF-8 system may have positively affected the toxicity reduction, in comparison with the drug alone.

Thus, the low cytotoxic activity of the materials was evident. Even after 48 h of treatment, no statistically significant toxic effects occurred in the concentrations tested on cell viability. The slow release of the drug by the BNZ@ZIF-8 system may have positively affected the toxicity reduction, in comparison with the drug alone.

ZIF-8 and BNZ@ZIF-8 system did not show significant cytotoxicity in the cells tested by the MTT method. ZIF-8 has been shown to be chemically inert, evidencing the fact that it may be a carrier as a pharmaceutical adjuvant. These results are in accordance with the data presented by Alves and Ren et al.^50,51^

Finally, the present work suggests that the BNZ@ZIF-8 system can be used as an antitrypanosomal API in pharmaceutical forms, since it has been shown to be safe due to its low cytotoxic potential at the times and concentrations tested. However, further studies are required in order to evaluate the in vitro and in vivo efficacy and cytotoxicity of these materials.

## Conclusions

The dialysis assay provided an estimative of the pH-dependent release from its own carrier (ZIF-8) and through the dialysis membrane. A faster release of BNZ in the system was observed at pH 4.5, when compared with pH 7.6. Furthermore, the BNZ concentration in both situations was lower than in the BNZ alone, keeping a prolonged release without oscillations and the burst effect observed in the drug alone. The drug release modulation could be proven by the model-independent method. By adjusting the release profiles, it was possible to suggest the mechanism of drug release from the BNZ@ZIF-8 system, which occurs depending on the pH tested.

## Experimental

### Materials

BNZ was acquired from the Pharmaceutical Laboratory of the State of Pernambuco (LAFEPE), lot 301045 (99.5%), while the ZIF-8, lot S45328-308, was donated by the Laboratório de Terras Raras of UFPE. Acetone (Modern Chemistry, lot 03196) and ultra-pure water (Mili-Q) were also used. Potassium phosphate (Vetec, lot 1007140) and sodium hydroxide (Sigma-Aldrich, batch SLBM7637) were used for the preparation of the dialysis test medium. The dialysis bags (10,000–12,000 Da) were obtained from Sartorius, Germany.

### Development of BNZ@ZIF-8 systems

The obtaining method used in the present study was based on the work of Horcajada et al.^36^ and optimized by our research group, which promoted the drug adsorption using a ZIF-8 suspension though an ex situ method^36^. The procedure used the molar ratio of BNZ@ZIF-8 1:1, based on the molecular weights of BNZ and ZIF, which are 260.25 and 229.61 g mol^−1^, respectively.

Initially the drug was solubilized with acetone in Erlenmeyer flasks with capacity of 250 mL, yielding an initial concentration of 20 μg mL^−1^. The BNZ solution was sonicated for 10 min in a Limp Sonic sonicator to ensure complete solubilization. At the end of the shaking, the ZIF-8 was suspended in acetone and added at the BNZ solution. Subsequently, the volume was filled with the respective solvent, submitting the mixture to intermittent stirring on Magnetic Stirrer MA089 Marconi with the aid of a magnetic bar for 4 days.

The supernatant was collected daily (0.33 mL, followed by solvent replenishment) and filtered through a hydrophobic filter with 0.22 μm pore aperture for further quantification by absorption spectroscopy in the Ultraviolet–Visible region (UV–Vis) with the aid of a previously performed calibration curve (Line equation: y = 0.0316x − 0.0003; R^2^ = 0.9994) for the construction of a BNZ incorporation curve into the ZIF-8 network. This process was used to determine the maximum incorporation efficiency (IE%), defined by Eq. 1.

1 $$ {\text{IE}}\% = \frac{{\left[ {{\text{BNZ}}\;{\text{theoretical}}\;{\text{concentration}}} \right] \times \left[ {\text{BNZ real concentration}} \right]}}{{\left[ {{\text{BNZ}}\;{\text{theoretical}}\;{\text{concetration}}} \right]}} \times 100 $$

At the end of the process, the material was centrifuged at 2000 RPM for 20 min in order to remove residual BNZ. The supernatant was discarded and the precipitate was washed with acetone. The same procedure was repeated twice. Then, the material was dry in a drying oven with 35 °C airflow (Shel Lab) until completely dry (about 4 h). Once dry, the material was used to quantify the EI% of the ZIF-8 network. Finally, the system presented an IE% = 38% (± 1.28%) and then it was called BNZ@ZIF-8. The BNZ@ZIF-8 system, composed by the association BNZ:ZIF-8 in 1:1 mol/mol ratio, presented itself as the best system among the tests carried out with other ratios in three levels (SM Fig. 1).

This entire procedure was done in triplicate and was performed in the absence of light due to the inherent photosensitivity of BNZ, as observed in previous studies. Due to the presence of acetone, the Erlenmeyer flasks were sealed in order to reduce the volatilization of the solvent. However, the volatilized content was constantly restored after verification of mass loss, based on the value of 0.79 g mL^−1^ for the acetone’s density^44^.

### Systems characterization

The materials were characterized by several analytical techniques to confirm the formation of the BNZ@ZIF-8 systems: Scanning Electron Microscopy (SEM), Polarized Light Microscopy (MLP), X-ray Diffraction (XRD), Thermal Analysis: Thermogravimetry (TG), Calorimetry Differential Scanning (DSC), Fourier transform infrared absorption spectroscopy (FTIR), Laser particle size and surface area analysis, and pore size and volume.

TG curves were performed using a Shimadzu Thermocouple, model TGA Q60, under a flowing nitrogen atmosphere of 50 mL min^−1^, sample mass (3.00 mg ± 0.05) for isolate BNZ and ZIF-8 and (6.00 mg ± 0.05) for platinum crucible Physical Mixtures (PM) and system in the temperature range of 25 to 1000 °C at the heating rate (β) of 10 °C min^−1^. Prior to the tests, thermobalance was checked with calcium oxalate. The BNZ and PM DSC curves were obtained using Shimadzu DSC-60 Calorimeter, interconnected to the Shimadzu TA-60WS software, with 50 mL min^−1^ nitrogen atmosphere and 10 °C min^−1^ heating rate in the range temperature of 25–500 °C. The samples were placed in a hermetically sealed aluminum sample holder with a mass of 3.0 ± 0.2 mg for BNZ and ZIF-8 and 6.0 ± 0.2 mg for PM and BNZ@ZIF-8 system. The determinations were performed in triplicate and indium and zinc were used to calibrate the temperature scale and the enthalpy response.

UV–Vis Spectra was obtained by scanning from 190 to 1000 nm. The ultraviolet spectrophotometer Shimadzu UV-2401 PC and quartz cuvettes with a cross section of 1 cm were used.

The FTIR spectra of BNZ, PM and BNZ@ZIF-8 were obtained using the PerkinElmer equipment (Spectrum 400) with an attenuated total reflectance (ATR) device of selenium crystal. The samples to be analyzed were transferred directly into the ATR device compartment. The results were obtained by scans from 4500 to 600 cm^−1^.

XRD analysis was performed on a Shimadzu XRD-7000 diffractometer, equipped with a copper anode at a scanning speed of 1.2° min^−1^, in the 2θ angle range from 5° to 45°. Basal spacing was calculated using the Bragg equation.

### In vitro dialysis release method under sink conditions

The in vitro release assay of BNZ was performed by dialysis method in order to define the modulated release profile by the BNZ@ZIF-8 system. Samples of 18.33 mg of BNZ were used in the dialysis studies, in which, if completely dissolved in a 250 mL of dissolution medium phosphate buffer release medium at pH 4.5 and 7.6, and 37 ± 0.5 °C, it would present a concentration of 73.32 μg mL^−1^, establishing, thus, the sink condition. The dissolution medium was chosen based on recent studies showing the modulation of the pH-dependent release of the drug from the ZIF-8 network^20,26,41^. A lower pH value was not used due to the rapid structural dissociation of ZIF-8, which would result in an extremely accelerated drug release^33^.

Sink condition was determined based on a quantitative solubility assay of BNZ performed by our research group where the maximum solubility in the dissolution media used (about 240 μg mL^−1^) was measured after 7 days of intermittent stirring. The experiments were performed under the suitable conditions described by Rohrs^52^ and the determination of Sink index, which employs dissolution medium volumes of 3 to 10 fold of the volume present in the saturated BNZ solution for sink condition.

During the experiment, 2 mL of the BNZ@ZIF-8 system formulation and the same volume of drug alone were both placed in dialysis bags (10.000–12.000 Da) (Sartorius, Germany). The dialysis membranes were immersed in 250 mL of dissolution medium, under continuous agitation at 75 RPM (Revolutions per minute). Aliquots (2 mL) from the external medium were collected at time intervals of 0.083, 0.25, 0.5, 0.75, 1, 2 h, followed by an exploratory collection until the drug release reaches at least 80%. The release medium was replenished at each collection to maintain the sink conditions. The amount of BNZ released was assessed by UV–Vis (BNZ absorbance wavelength = 323 nm) (Fig. 1).

In order to compare the dissolution profiles between BNZ and the system, the nonlinear method that calculates the value of ƒ2, known as similarity factor, was used. The ƒ2 metric is a function of the reciprocal of mean square root transform of the sum of square distances. The ƒ2 can be obtained through Eq. 2^53^.

2 $$ f2 = 50 \times \log \left\{ {\sqrt {\left[ {1 + \left( \frac{1}{n} \right)\mathop \sum \limits_{t = 1}^{n} \left( {Rt - Tt} \right)^{2} } \right]^{ - 0.5} } \times 100} \right\} $$

where *n* is the number of collection times considered for calculation purposes; Rt is the percentage value dissolved at time *t* obtained with the reference or comparator curve; and Tt is the percent dissolved value of the test curve, or the altered formulation at time *t*.

### In vitro evaluation of cell viability and cytotoxicity by MTT assay

For in vitro cellular cytotoxicity assay, whole human blood (5 mL) from healthy and non-smoking volunteers was obtained by venipuncture (with informed consent and approved by the National Ethics Committee. Reference number: 30667014.5.0000.5208). Peripheral blood mononuclear cells (PBMC) were obtained from whole blood by Ficoll-quantum method (Ficoll-Paque Plus)^54,55^. Human PBMC were cultured at 5.1 × 10^3^ cells/well in a 96-wells plate containing Dulbecco MEM culture medium (DMEM) supplemented with 10% phosphate buffered saline.

The cells were incubated with BNZ and ZIF-8 on 3 different conditions: (1) ZIF-8 at 150 μg mL^−1^ concentration, (2) BNZ at 1, 10 and 100 µg mL^−1^ concentrations and (3) BNZ@ZIF-8 system under the same conditions as the drug alone. Cells were incubated for 24 and 48 h at 37 °C in 5% CO_2_. Then the culture medium was replaced by 100 µL of fresh DMEM with 10 µL of tetrazolium dye 3-(4,5-dimethylthiazol-2-yl)-2,5-diphenyltetrazolium bromide (MTT) (5 mg mL^−1^ in phosphate buffer) per well, proceeding with the incubation for 4 h at 37 °C in the dark in 5% CO_2_. Untreated cells, MTT and buffer-phosphate were used as negative control and the medium alone (without cells), MTT and buffer-phosphate were used as the positive control. Dead cells (0.1–1% Triton-X), MTT and buffer-phosphate were used as the blank. Finally, absorbance (at 570 nm) was recorded by ELISA microplate reader.

Regarding the statistical analysis, each trial was performed in biological duplicates and technical triplicates, and the results were expressed as mean ± standard deviation. Statistical analysis was performed using GraphPad Prism software version 5.0. *p* values less than 0.05 were considered statistically significant. The results were compared by analysis of variance (ANOVA), followed by Dunn’s multiple comparison test and Student’s *t* test. The concentration required for the half maximal inhibitory concentration (IC 50) was graphically estimated by linear regression analysis, and correlation indices were calculated using the Pearson (r) coefficient.

### Ethical statement

The author declares that all the experiment protocol involving humans is in accordance to guidelines of the National Research Ethics Committee (CONEP) of the National Health Council—Brazil Ministry of Health (CNS / MS). The blood used in the MTT assay was collected from volunteers after signing the informed consent form, registered under reference number: 30667014.5.0000.5208.

## Supplementary information

Supplementary Figure 1
