# Supplementary material for: ZIF-8 as a promising drug delivery system for benznidazole: development, characterization, in vitro dialysis release and cytotoxicity
Source: Sci Rep. 2020 Oct 8;10:16815. doi: 10.1038/s41598-020-73848-w (PMC7545170; doi:10.1038/s41598-020-73848-w)
Supplement: Supplementary file 1 — Supplementary Figure 1 [file 41598_2020_73848_MOESM1_ESM.docx]

*Supplementary Material*

**ZIF-8 as a Promising Drug Delivery System for Benznidazole: Development, Characterization, *in vitro* Dialysis Release and Cytotoxicity**

**Leslie Raphael de Moura Ferraz^1*^, Alinne Élida Gonçalves Alves Tabosa^1^, Débora Dolores Souza da Silva Nascimento^1^, Aline Silva Ferreira^1^, Victor de Albuquerque Wanderley Sales^1^, José Yago Rodrigues Silva^2^, Severino Alves Junior^2^, Larissa Araújo Rolim^3^, Jorge José de Souza Pereira^4^, Pedro Jose Rolim-Neto^1^**

^1^Laboratório de Tecnologia dos Medicamentos – Department of Pharmaceutical Sciences, Federal University of Pernambuco, Av. Prof. Arthur de Sá, s/n, Cidade Universitária, 50740-521 Recife - PE, Brazil.

^2^Laboratório de Terras Raras – Departamento de Química Fundamental, Federal University of Pernambuco, Av. Jornalista Aníbal Fernandes, s/n - Cidade Universitária, 50740-560, Recife-PE, Brazil.

^3^Central Analítica - Colegiado de Ciências Farmacêuticas, Federal University of Vale do São Francisco, Av. José de Sá Maniçoba, s/n, Centro, 56304-917, Petrolina - PE, Brazil.

^4^Laboratory of Immunopathology Keizo Asami (LIKA), Federal University of Pernambuco, Recife, Brazil

*Corresponding Author: prof.raphaelferraz@gmail.com

**Development of BNZ@ZIF-8 systems**

In order to further optimize the method of obtaining the systems using acetone, different molar ratios (1:3, 3:1 and 6:1 mol/mol) were evaluated in order to identify the ratio that presented the highest incorporation efficiency (IE%). However, the IE% values were all below that presented by the molar ratio of 1:1 (0; 21.07 and 23.57, respectively), as seen in SM Figure 1.

**SM Figure 1.** Incorporation curves of BNZ into the ZIF-8 network obtained in acetone with different molar ratios (the dashed line refers to initial concentration, IE% = incorporation efficiency).
